# Long-Term Propranolol Treatment Induces Anxiolytic-like Effects and Alters Hypothalamic Noradrenergic, Serotonergic and GABAergic Neurotransmission

**DOI:** 10.3390/ijms27219755

**Published:** 2026-10-30

**Authors:** Adrianna Witczyńska, Łukasz Fijałkowski, Kamilla Blecharz-Klin, Katarzyna Ziętal, Justyna Pyrzanowska, Agnieszka Piechal, Ilona Joniec-Maciejak, Ewa Machaj, Agnieszka Jastrzębska, Jacek Nowaczyk, Dagmara Mirowska-Guzel, Alicja Nowaczyk

**Affiliations:** 1 Department of Organic Chemistry, Faculty of Pharmacy, Collegium Medicum in Bydgoszcz, Nicolaus Copernicus University, 87-100 Toruń, Poland; adrianna.witczynska@doktorant.umk.pl (A.W.); l.fijalkowski@cm.umk.pl (Ł.F.); 2 Department of Experimental and Clinical Pharmacology, Centre for Preclinical Research and Technology CePT, Medical University of Warsaw, Banacha 1b, 02-097 Warsaw, Poland; kamilla.blecharz-klin@wum.edu.pl (K.B.-K.); katarzyna.zietal@wum.edu.pl (K.Z.); justyna.pyrzanowska@wum.edu.pl (J.P.); agnieszka.piechal@wum.edu.pl (A.P.); ilona.joniec-maciejak@wum.edu.pl (I.J.-M.); ewa.machaj@wum.edu.pl (E.M.); dagmara.mirowska-guzel@wum.edu.pl (D.M.-G.); 3 Central Laboratory of Laboratory Animals, Centre for Preclinical Research and Technology (CePT), Medical University of Warsaw, Banacha 1b, 02-097 Warsaw, Poland; agnieszka.jastrzebska@wum.edu.pl; 4 Faculty of Chemistry, Nicolaus Copernicus University, 7 Gagarina St., 87-100 Toruń, Poland; jacek.nowaczyk@umk.pl

**Keywords:** propranolol, CNS, anxiety-related behavior, hypothalamus, GABA, noradrenaline, serotonin, monoamine turnover, neurotransmission, neurochemical modulation, neuropharmacology

## Abstract

Propranolol is a non-selective β-adrenergic receptor antagonist widely used in cardiovascular medicine and anxiety-related conditions; however, its central neurochemical mechanisms remain incompletely understood. This study investigated the effects of long-term propranolol administration on anxiety-like behavior and hypothalamic neurotransmission in rats. Adult male Sprague–Dawley rats received propranolol in drinking water at doses of 1, 2.5, or 5 mg/kg b.w./day for 33 days. Behavioral effects were assessed using the Open Field Test (OF) and Elevated Plus Maze (EPM). Hypothalamic concentrations of amino acids, e.g., γ-aminobutyric acid (GABA) as well as monoamines, and their metabolites were determined by high-performance liquid chromatography with electrochemical detection (HPLC-ED). Long-term propranolol treatment induced a significant anxiolytic-like effect, reflected by increased exploration of open arms in the EPM, without affecting locomotor activity in the OF. Neurochemical analyses revealed a significant increase in hypothalamic GABA levels and a marked reduction in noradrenergic turnover, as indicated by decreased MHPG/NA ratios. Propranolol also increased hypothalamic serotonin concentrations, whereas dopaminergic turnover remained unchanged. These findings demonstrate that long-term propranolol administration produces neurochemical changes within the hypothalamus involving noradrenergic, serotonergic, and GABAergic signaling pathways. The results suggest that the anxiolytic-like effects of propranolol extend beyond β-adrenergic receptor blockade and may be mediated by coordinated modulation of multiple neurotransmitter systems involved in stress regulation and emotional processing. The study provides new evidence linking chronic propranolol exposure to selective hypothalamic neurochemical remodeling associated with reduced anxiety-like behavior.

## 1. Introduction

Anxiety disorders are among the most common psychiatric disorders worldwide. They affect millions of people and markedly reduce quality of life. Their development is associated with disturbances in several neurotransmitter systems. The noradrenergic, serotonergic, and GABAergic systems are considered particularly important in the regulation of emotional responses and stress adaptation [1,2,3].

Noradrenaline is one of the principal mediators of the stress response. Increased noradrenergic activity promotes arousal, vigilance, and autonomic activation. Excessive stimulation of this system is commonly observed in anxiety-related disorders [1,2,4,5].

GABA exerts a predominantly inhibitory influence on neuronal activity and represents the principal inhibitory neurotransmitter in the central nervous system (CNS). By counterbalancing excitatory neurotransmission and constraining excessive neuronal activation, GABAergic signaling is essential for maintaining neuronal network stability and regulating emotional and stress-related responses. Impairment of GABAergic transmission may disrupt the excitation–inhibition balance within neural circuits involved in anxiety and stress processing and has been consistently associated with increased anxiety-like behavior and reduced adaptive responses to stress [2,3,6,7].

Serotonin is a key neuromodulatory system involved in the integration of emotional processing, stress responses, and behavioral adaptation. Perturbations in serotonergic neurotransmission can alter the neural circuits underlying anxiety and stress coping, with sustained dysregulation being associated with heightened vulnerability to stress-related disorders and reduced resilience to adverse environmental challenges [1,2,8].

Propranolol is a non-selective β-adrenergic receptor antagonist used for decades in the treatment of cardiovascular diseases. Its high lipophilicity enables efficient penetration of the blood–brain barrier. As a result, propranolol exerts both peripheral and CNS effects [4,5]. Clinical observations and experimental studies indicate that propranolol reduces anxiety symptoms, modifies emotional responses to stressful stimuli, and affects memory processes associated with fear and stress [9,10].

The central mechanisms responsible for these effects remain incompletely understood. Although most studies have focused on β-adrenergic receptor blockade, increasing evidence indicates that the pharmacological profile of propranolol is considerably more complex than its canonical β-adrenergic effects alone [9,11,12].

Propranolol is administered as a racemic mixture of R-(+)- and S-(−)-propranolol. Because biological targets are inherently chiral, individual enantiomers may differ in their affinity toward molecular targets, pharmacokinetic behavior, and biological activity. Such stereoselective effects have been described for numerous CNS-active drugs and may significantly influence therapeutic outcomes [13,14,15,16]. Although stereoselectivity is relevant to the overall pharmacological profile of propranolol, the present study investigated the effects of the racemic preparation and was not designed to distinguish the contributions of individual enantiomers.

Growing attention has also been devoted to the physicochemical properties of propranolol and their relationship to biological activity. Chromatographic, crystallographic, and in silico approaches have provided valuable information regarding bioavailability, membrane interactions, target accessibility, and ligand–protein recognition. Analyses of propranolol-containing structures deposited in the Protein Data Bank (PDB) demonstrated interactions with both canonical and non-canonical protein targets, highlighting the importance of molecular geometry, lipophilicity, and stereochemistry in determining ligand binding and pharmacological activity [17,18,19,20].

These observations suggest that molecular properties contribute substantially to the overall pharmacological profile of propranolol and may influence its interactions with biological targets. The hypothalamus is one of the major brain structures involved in the regulation of stress responses. It integrates endocrine, autonomic, and behavioral signals. It also coordinates activation of the hypothalamic–pituitary–adrenal axis [21]. This neuroendocrine response is tightly regulated by interacting excitatory and inhibitory neurotransmitter systems within hypothalamic circuits. In particular, noradrenergic projections strongly modulate hypothalamic activity during stress, whereas GABAergic neurons provide inhibitory control over these circuits and help prevent excessive activation of stress pathways [3,21,22,23]. Proper communication between these systems is necessary for the maintenance of emotional homeostasis.

Serotonergic signaling is also critically involved in the regulation of hypothalamic stress responses, contributing to the modulation of neuroendocrine function and behavioral adaptation to aversive stimuli [1,2,8]. Increasing evidence indicates that serotonergic, noradrenergic, and GABAergic systems form an interconnected network through which stress-related neural activity is dynamically regulated [1,2,3,9]. Given the ability of propranolol to influence central noradrenergic signaling, interactions with these interconnected neurotransmitter systems may contribute to its broader neuropharmacological profile and could help explain central effects that are not fully accounted for by β-adrenergic receptor blockade alone.

The hypothalamus was selected as the primary brain region for analysis because of its central role in integrating neural, neuroendocrine, autonomic, and stress-related responses. Recent advances in neuroscience have substantially expanded our understanding of the hypothalamus, revealing remarkable molecular and functional heterogeneity among its neuronal populations and their capacity to integrate hormonal and neural signals involved in energy homeostasis, stress, and other physiological functions [24]. Importantly, recent experimental evidence has demonstrated that specific neuronal populations within the lateral hypothalamus encode anxiogenic stimuli and contribute to adaptive behavioral responses under threatening conditions, providing direct evidence for the involvement of hypothalamic circuits in the regulation of anxiety-related behavior [25]. Furthermore, human neuroimaging evidence has shown that functional connectivity of specific hypothalamic nuclei is associated with individual differences in prolonged stress, with nucleus-specific connectivity providing more informative associations with stress than connectivity assessed at the level of the whole hypothalamus [26].

These findings support the view of the hypothalamus not merely as a neuroendocrine control center, but as a highly heterogeneous integrative hub linking neuroendocrine, autonomic, stress-related, and emotional processes. This is particularly relevant to the present study, as serotonergic, dopaminergic, noradrenergic, and GABAergic signaling contributes to the regulation of hypothalamic responses to stress and emotional states. Therefore, characterization of hypothalamic neurochemistry provides a biologically relevant approach for investigating potential central neurochemical adaptations induced by chronic propranolol administration and their relationship to anxiety-like behavior.

Despite the extensive clinical use of propranolol, relatively little is known about its effects on hypothalamic neurotransmission following chronic administration. In particular, although previous studies have demonstrated that propranolol may influence individual central neurotransmitter systems, the effects of prolonged treatment on serotonergic, dopaminergic, noradrenergic, and GABAergic signaling within the hypothalamus remain insufficiently characterized. Previous studies have generally focused on individual neurotransmitter systems or specific aspects of propranolol action, and the combined effects of chronic propranolol administration on these major neurotransmitter systems within the same experimental paradigm have not been comprehensively examined. Moreover, the dose-specific effects of chronic propranolol exposure on hypothalamic neurochemistry, including monoamine turnover and GABAergic activity, remain insufficiently understood.

We hypothesized that prolonged propranolol administration would be associated with changes in anxiety-like behavior and hypothalamic neurochemical parameters, and that the magnitude and pattern of these changes might differ between treatment doses. A better understanding of these changes may help elucidate the neurochemical mechanisms underlying the central effects of propranolol, including its anxiolytic-like activity.

Therefore, the present study adopted a multisystem approach to investigate the effects of long-term propranolol administration at three different doses on anxiety-like behavior and hypothalamic neurochemistry in rats. Particular attention was given to the concentrations of the amino acid neurotransmitter GABA and the monoamines noradrenaline, serotonin, and dopamine, together with indices of monoaminergic turnover. By assessing these neurotransmitter systems under identical experimental conditions, the study was designed to provide an integrated characterization of the hypothalamic neurochemical effects of chronic propranolol exposure and to determine whether these effects are dose-specific, thereby identifying potential neurochemical mechanisms underlying its anxiolytic-like activity. The findings are intended to identify candidate neurochemical correlates for further investigation, rather than to establish causal mechanisms underlying the behavioral effects of propranolol.

## 2. Results

### 2.1. Effects of Long-Term Propranolol Treatment on Body Weight and Hemodynamic Parameters

**Figure 1:**
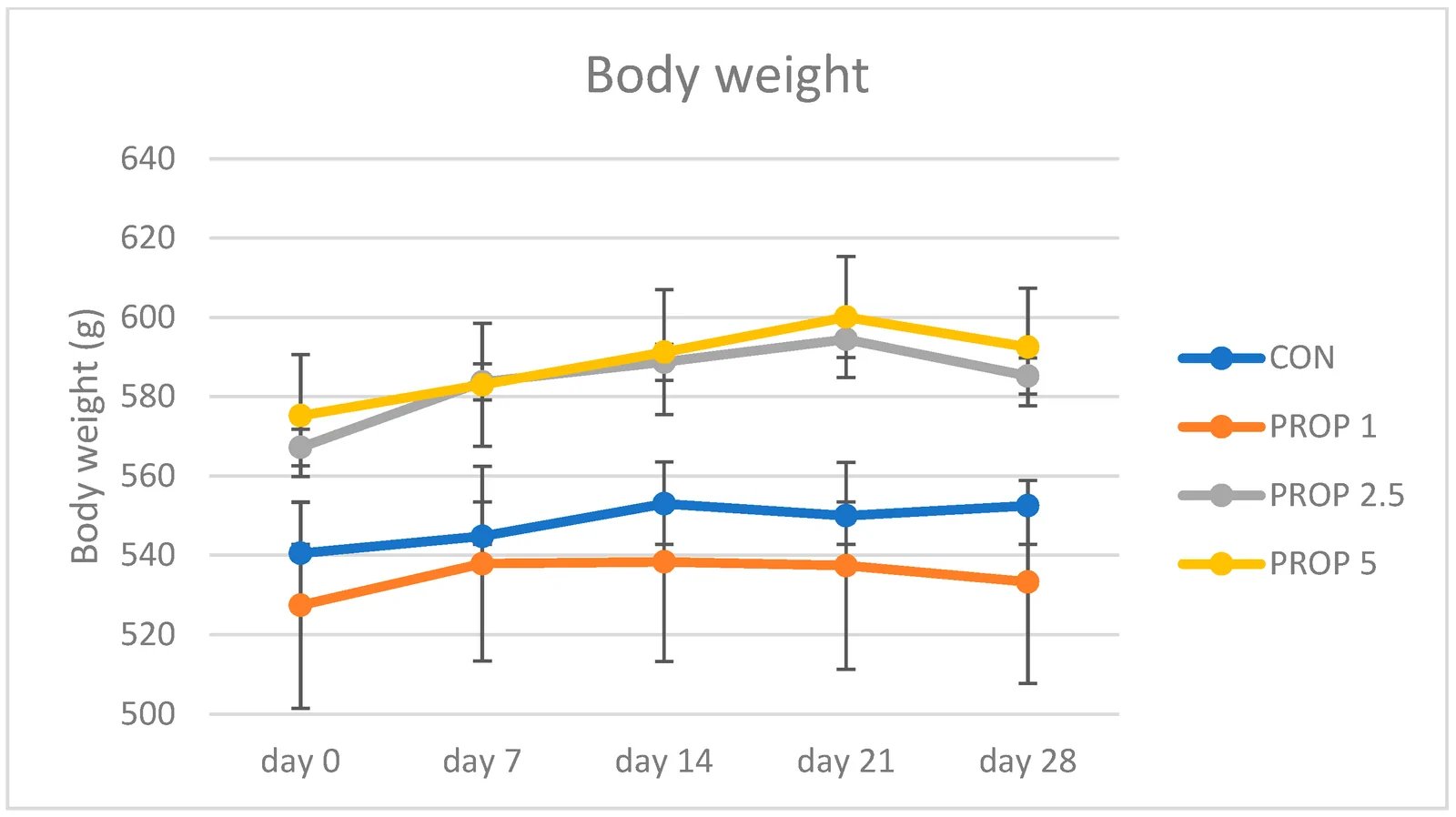
Time course of body weight in control and propranolol-treated rats (n = 10 per group).

Body weight was monitored throughout the experimental period (days 0, 7, 14, 21, and 28) (Figure 1). No statistically significant differences in body weight were observed between the experimental groups at baseline (day 0) (F(3,36) = 1.29, *p* = 0.29). Although body weight changed over the course of the experiment, chronic propranolol treatment did not result in statistically significant differences in body weight between the control and propranolol-treated groups at any of the assessed time points (*p* > 0.05). Mean cage-level water consumption did not differ significantly among the control and propranolol-treated groups over the experimental period (one-way ANOVA, F(3,4) = 1.050, *p* = 0.462; Supplementary Table S1). Estimated daily propranolol exposure is presented in Supplementary Table S2. Given the limited number of cages per group, this finding should be interpreted cautiously.

All animals remained in good general condition, and no treatment-related adverse effects were noted.

**Figure 2:**
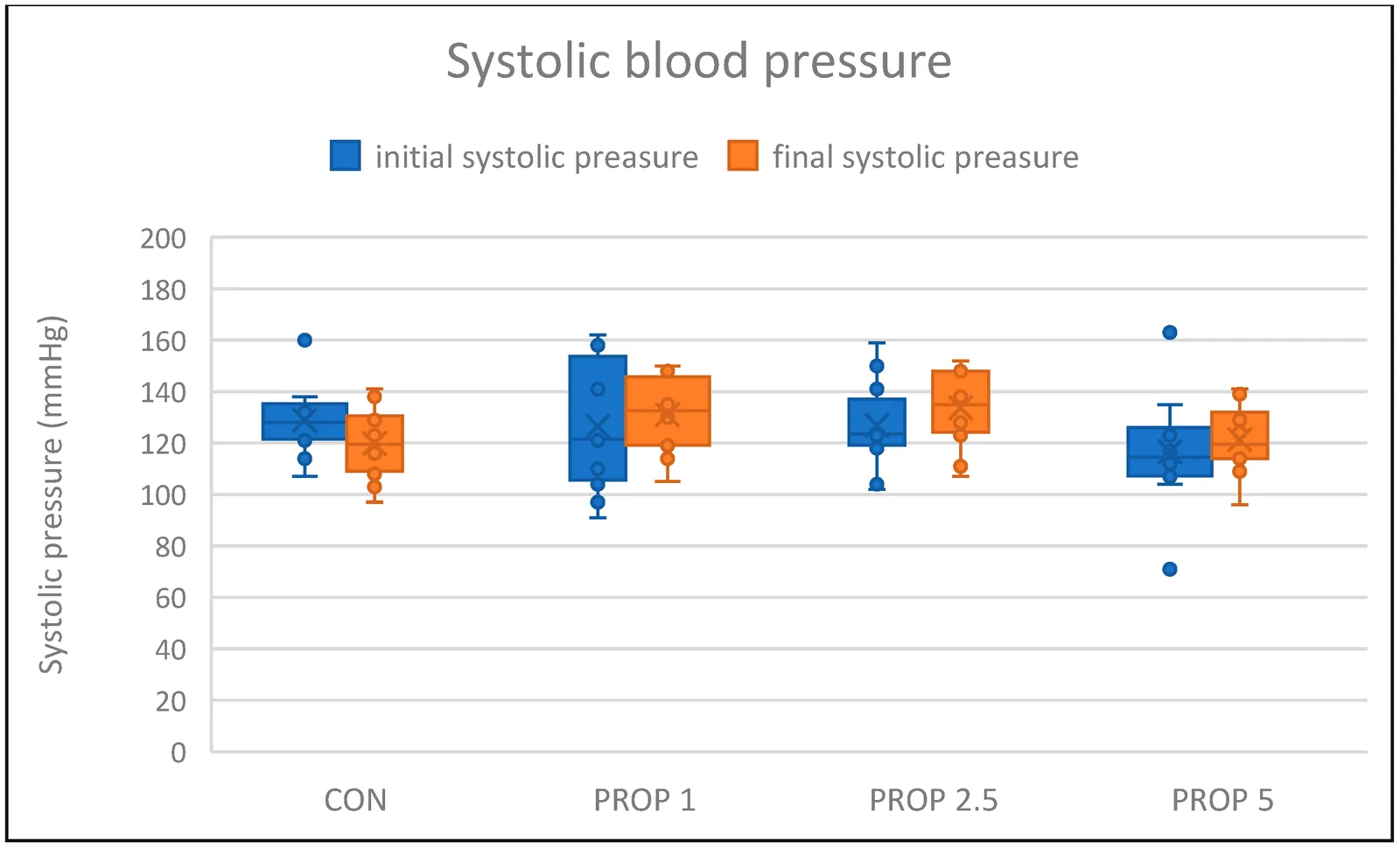
Changes in systolic blood pressure between baseline and the end of long-term propranolol treatment (n = 10 per group). Horizontal line—median, ×—mean.

**Figure 3:**
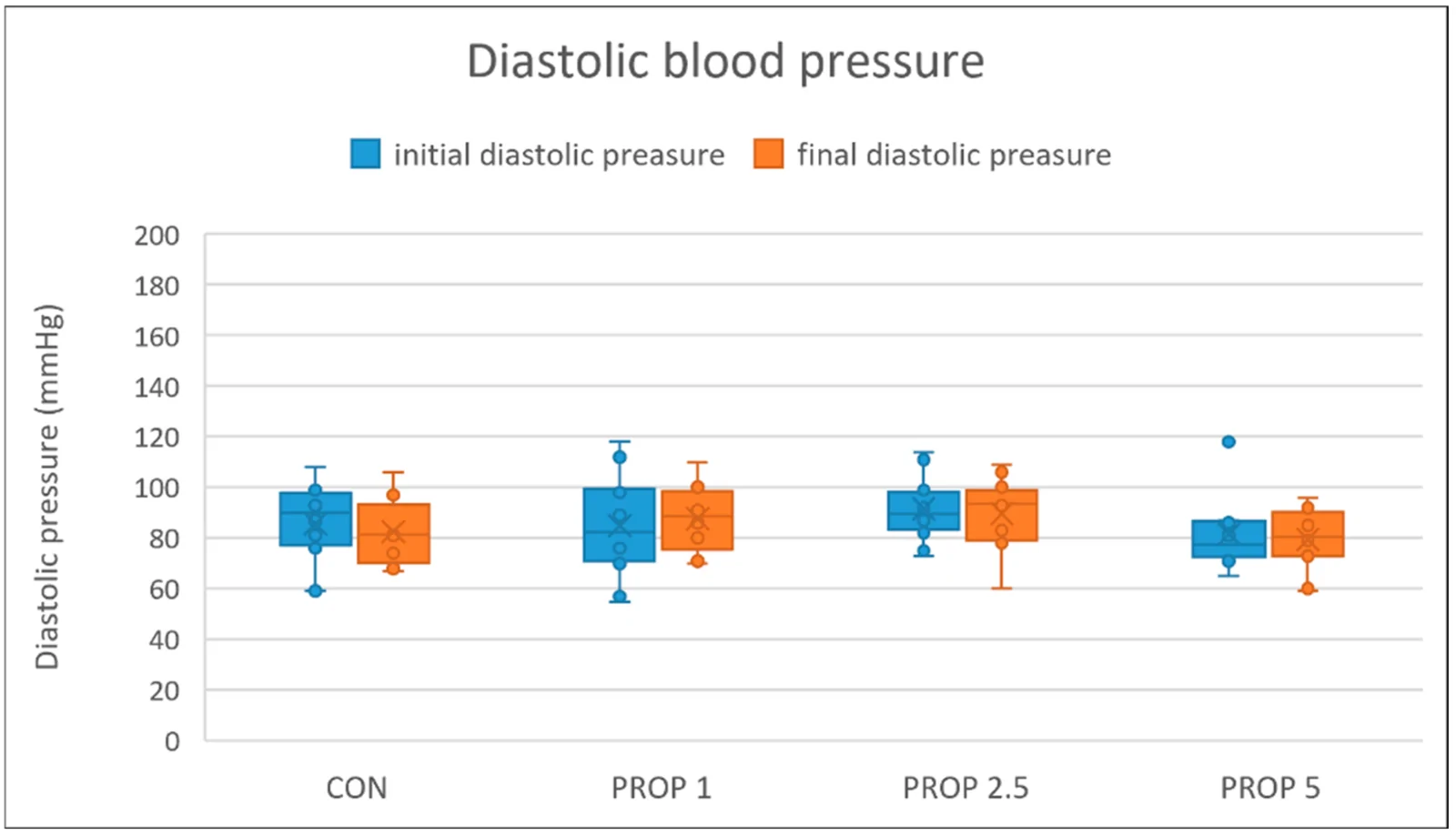
Changes in diastolic blood pressure between baseline and the end of long-term propranolol treatment (n = 10 per group). Horizontal line—median, ×—mean.

**Figure 4:**
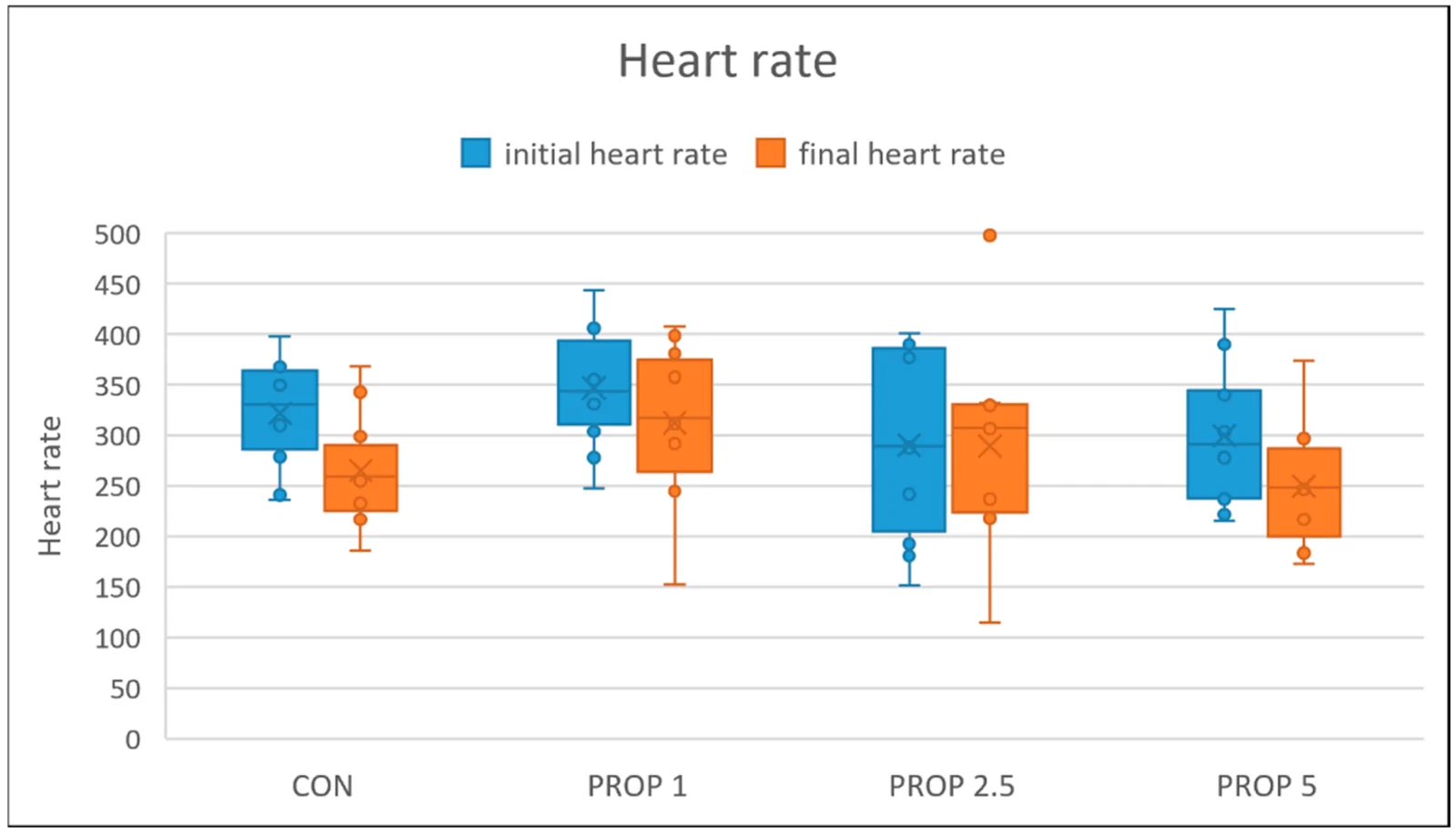
Heart rate measured at baseline and after chronic propranolol treatment in control and treated rats (n = 10 per group). Horizontal line—median, ×—mean.

Similarly, long-term propranolol treatment did not significantly affect systolic blood pressure (Figure 2), diastolic blood pressure (Figure 3), or heart rate (Figure 4). Hemodynamic parameters remained within the physiological range in all experimental groups, with no significant differences between propranolol-treated and control animals.

These findings indicate that prolonged administration of propranolol at doses of 1, 2.5, and 5 mg/kg b.w./day did not induce marked cardiovascular alterations under the experimental conditions used in the present study. Therefore, the behavioral and neurochemical effects observed in subsequent analyses are unlikely to be secondary to changes in general physiological status.

### 2.2. Effects of Propranolol in the Open Field Test

Long-term propranolol administration did not significantly affect spontaneous locomotor activity or exploratory behavior in the Open Field Test (Table 1).

No significant differences were observed between propranolol-treated animals and control rats in any of the analyzed parameters.

Propranolol treatment did not significantly affect climbing frequency at any dose (F(3,36) = 1.15, *p* = 0.34). The frequency of entries into the central zone remained unchanged in all treatment groups (F(3,36) = 0.97, *p* = 0.96). Similarly, the time spent in the central zone did not differ significantly between groups (F(3,36) = 0.63, *p* = 0.60).

Locomotor activity, assessed by total distance traveled (F(3,36) = 1.6, *p* = 0.21), movement velocity (F(3,36) = 1.36, *p* = 0.27), and time spent in movement (F(3,36) = 1.06, *p* = 0.38), was comparable across all experimental groups. Given that chronic propranolol treatment did not alter locomotor activity or exploratory behavior in the Open Field Test, the behavioral effects observed in the Elevated Plus Maze are unlikely to reflect nonspecific motor impairment or sedation.

Overall, these findings indicate that chronic propranolol treatment at doses of 1, 2.5, and 5 mg/kg/day did not impair locomotor activity or exploratory behavior.

### 2.3. Effects of Propranolol in the Elevated Plus Maze

Given that long-term propranolol administration did not affect locomotor activity or exploratory behavior in the OF, the behavioral effects observed in the EPM are unlikely to be attributable to nonspecific motor impairment or sedation. In the EPM, propranolol-treated rats showed increased exploration of the open arms, reflected by changes in several parameters commonly associated with reduced anxiety-like behavior. These effects were most pronounced at the highest propranolol dose, indicating a dose-related enhancement of open-arm exploration and supporting an anxiolytic-like profile of long-term propranolol treatment.

**Figure 5:**
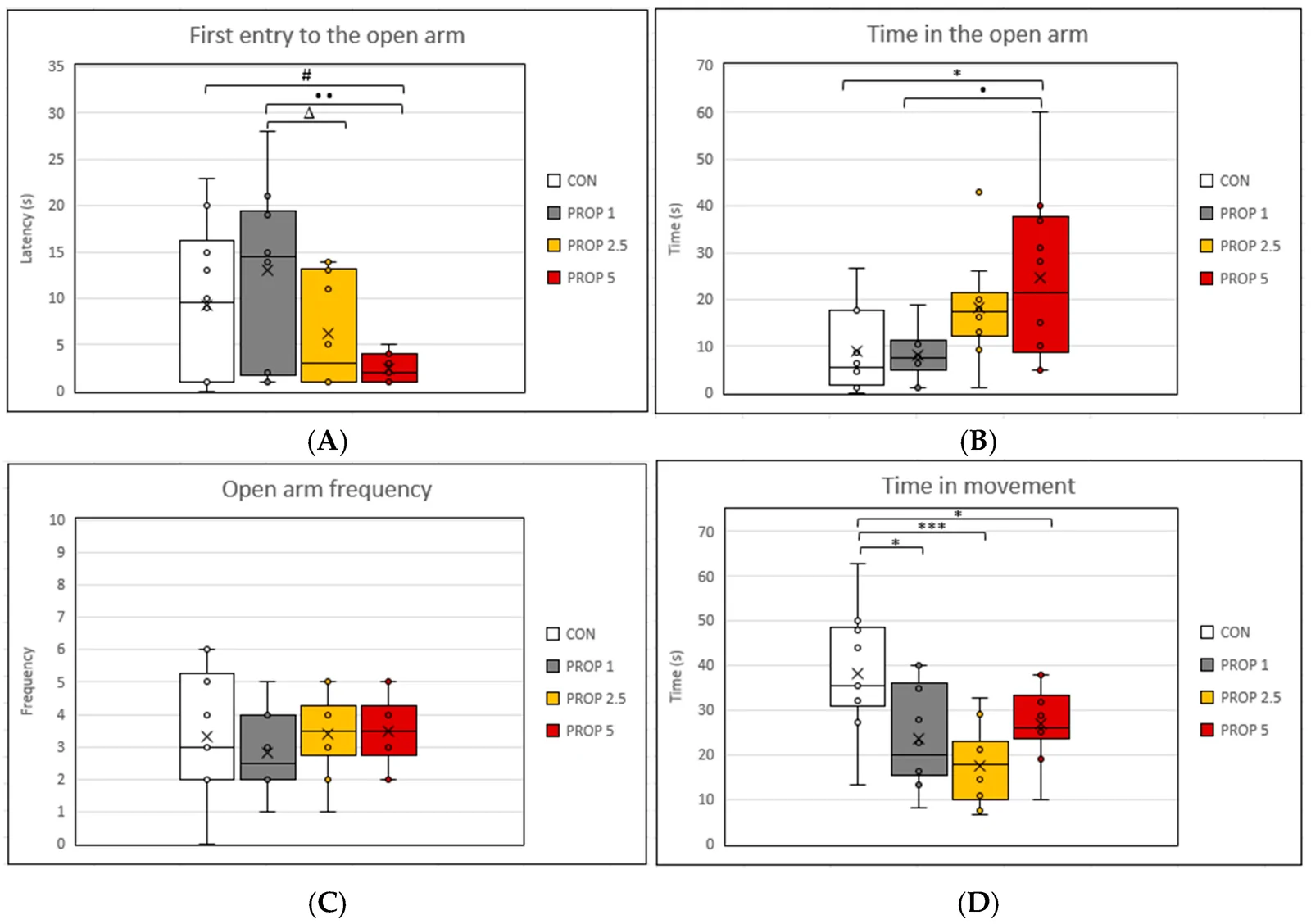
Effects of long-term propranolol administration on anxiety-like behavior in the Elevated Plus Maze (EPM) in rats receiving propranolol in drinking water at doses of 1, 2.5, or 5 mg/kg b.w./day for 33 consecutive days (n = 10 per group). (**A**) Latency to the first entry into an open arm; (**B**) time spent in the open arms; (**C**) frequency of open-arm entries; and (**D**) time spent in movement. Data are presented as box-and-whisker plots showing median, interquartile range, minimum and maximum values; × indicates the mean. Statistical significance was determined using ANOVA followed by the Newman–Keuls post hoc test. * *p* < 0.05, *** *p* < 0.005 groups vs. the control group (NK); ^Δ^ *p* < 0.05 PROP 1 vs. PROP 2.5, vs. PROP 1 (NK); ^#^ *p* < 0.05 vs. PROP 5. ^●^ *p* < 0.05, ^●●^ *p* < 0.01 PROP 1 vs. PROP 5, (NK). Horizontal line—median, ×—mean.

In the EPM, long-term propranolol administration altered anxiety-like behavior, as indicated by changes in the latency to the first open-arm entry and time spent in the open arms, but not in the frequency of open-arm entries defined as the placement of all four paws inside an arm of the maze (Figure 5A–C). Time spent in movement was also significantly affected by propranolol treatment (Figure 5D).

The anxiolytic-like effect was reflected by a reduction in the latency to the first open-arm entry (F(3,36) = 4.22, *p* = 0.01; Figure 5A) and an increase in the time spent in the open arms (F(3,36) = 4.53, *p* = 0.008; Figure 5B). In contrast, the frequency of open-arm entries did not differ significantly between the groups (F(3,36) = 0.48, *p* = 0.70; Figure 5C).

Propranolol treatment also significantly affected the time spent in movement in the Elevated Plus Maze, with control animals spending more time in movement than propranolol-treated rats (F(3,36) = 6.36, *p* = 0.001; Figure 5D). However, this reduction in movement was accompanied by a shorter latency to enter the open arms and increased time spent in the open arms, while the frequency of open-arm entries remained unchanged. Together with the absence of marked alterations in locomotor activity in the OF, these findings support an anxiolytic-like effect of long-term propranolol treatment rather than a nonspecific impairment of motor function.

### 2.4. Effects of Propranolol on Hypothalamic Effects Amino Acid Neurotransmitters

Long-term propranolol administration significantly increased hypothalamic GABA levels (F(3,28) = 6.11, *p* = 0.002), while no significant changes were observed in the levels of the other amino acids analyzed (Table 2).

The strongest effect was observed in the PROP 1 group, in which GABA concentration increased from 844.39 ± 88.52 ng/mg in control animals to 1156.04 ± 53.32 ng/mg (*p* < 0.005). A significant increase was also detected in the PROP 2.5 group compared to the control group (*p* < 0.05). Although GABA levels remained elevated in the PROP 5 group, the effect was less pronounced than in the lower-dose groups and was significantly lower than that observed in the PROP 1 group (*p* < 0.05).

These findings indicate that long-term propranolol treatment significantly increases hypothalamic GABA levels at low and intermediate doses.

### 2.5. Effects of Propranolol on Hypothalamic Monoamine Levels, Metabolites, and Turnover

Long-term propranolol administration produced selective changes in hypothalamic monoaminergic neurotransmission, as assessed by the concentrations of individual monoamines and their metabolites (Table 3).

Monoamine turnover indices were derived from metabolite-to-neurotransmitter ratios and are reported in Table 4.

Noradrenergic system. One-way ANOVA revealed a significant effect of treatment on hypothalamic noradrenaline (NA) levels (F = (3,28) = 3.96, *p* = 0.018). Post hoc analysis showed that NA levels were significantly higher in rats treated with propranolol at 1 and 2.5 mg/kg b.w./day than in controls (*p* < 0.05), whereas the 5 mg/kg b.w./day group did not differ significantly from controls (Table 3). MHPG concentrations were not significantly affected by treatment (Table 3). A significant effect of treatment was also observed for the MHPG/NA ratio (F = (3,28) = 3.92, *p* = 0.019). Post hoc analysis demonstrated significantly lower MHPG/NA ratios in all propranolol-treated groups compared with controls (*p* < 0.05; Table 4).

Dopaminergic system. No significant effect of treatment was observed for hypothalamic dopamine (DA) levels or for the concentrations of its metabolites DOPAC, HVA, and 3-MT (Table 3). Similarly, the DOPAC/DA and HVA/DA ratios did not differ significantly among the experimental groups (Table 4), indicating no significant alteration of dopaminergic turnover.

Serotonergic system. One-way ANOVA revealed a significant effect of treatment on hypothalamic serotonin (5-HT) levels (F = (3,28) = 3.22, *p* = 0.038). Post hoc analysis showed that 5-HT levels were significantly higher in rats receiving propranolol at 1 mg/kg b.w./day than in controls (*p* < 0.05) (Table 3). 5-HIAA concentrations were not significantly affected by treatment. Likewise, no significant differences were observed in the 5-HIAA/5-HT ratio (Table 4), indicating that serotonergic turnover was not significantly altered.

Overall, long-term propranolol treatment selectively affected hypothalamic noradrenergic and serotonergic neurotransmission, as evidenced by increased NA and 5-HT levels and a significant reduction in the MHPG/NA turnover index, while dopaminergic neurotransmission and serotonergic turnover remained unaffected.

## 3. Discussion

The hypothalamus occupies a central position in the regulation of stress and emotional behavior, integrating neural signals with autonomic and neuroendocrine responses [21,22,23,27]. Its functional activity is shaped by interactions among several neurotransmitter systems, including noradrenergic, GABAergic, serotonergic, and dopaminergic signaling [2,3,28]. Disruption of this neurochemical balance may alter stress responsiveness and contribute to anxiety-related behavior [1,2,28,29].

Within this framework, the present study demonstrates that long-term propranolol treatment was associated with an anxiolytic-like behavioral profile accompanied by selective neurochemical changes in the hypothalamus. Propranolol increased open-arm exploration in the EPM, without producing significant alterations in locomotor or exploratory activity in the OF. These behavioral effects were accompanied by increased hypothalamic GABA concentrations, reduced MHPG/NA turnover, and a selective increase in 5-HT levels, whereas dopaminergic measures remained unchanged. Together, these findings suggest that prolonged β-adrenergic receptor blockade may induce coordinated but pathway-selective adaptations within hypothalamic neurotransmitter systems.

### 3.1. Behavioral Effects of Long-Term Propranolol Treatment

The behavioral profile observed in the EPM is consistent with reduced anxiety-like behavior. Propranolol-treated animals showed a shorter latency to enter the open arms and spent more time in these exposed compartments, whereas the frequency of open-arm entries remained unchanged. Increased exploration of the open arms is commonly interpreted as reduced avoidance of anxiogenic environmental conditions and is therefore considered indicative of an anxiolytic-like response [29,30,31].

Anxiety-related behavior is regulated by interactions among multiple neurotransmitter systems, with noradrenergic signaling playing an important role in arousal and responses to threatening stimuli. Excessive noradrenergic activity may promote hypervigilance and increased responsiveness to stress, while dysregulation of noradrenergic circuits has been implicated in anxiety-related psychopathology [1,2,28]. The locus coeruleus–noradrenergic system is also closely involved in the regulation of stress adaptation and resilience [4,25].

Propranolol differs from classical anxiolytic agents because its primary pharmacological target is the β-adrenergic receptor. Nevertheless, its high lipophilicity facilitates penetration of the blood–brain barrier, allowing it to affect central as well as peripheral processes [9,10]. Previous clinical and experimental studies have reported effects of propranolol on anxiety symptoms, stress-related physiological responses, emotional memory, and fear-related processes [9,11,12]. The present findings extend these observations by demonstrating an anxiolytic-like behavioral profile following prolonged administration of relatively low doses.

An important consideration is that propranolol did not significantly alter locomotor activity in the OF. Total distance traveled, movement velocity, and movement time remained comparable among the experimental groups. Thus, the increased open-arm exploration observed in the EPM is unlikely to be explained by generalized motor suppression. This distinction is particularly relevant when interpreting behavioral effects of a drug with both cardiovascular and central actions, as reduced locomotor activity could otherwise confound the assessment of anxiety-related behavior [29,30,31,32].

The behavioral effects observed in the EPM were accompanied by neurochemical alterations within the hypothalamus. Propranolol increased GABA concentrations, reduced the MHPG/NA turnover index, and selectively altered hypothalamic serotonin levels. Because the hypothalamus integrates autonomic, endocrine, and behavioral responses to stress [21,22,27], neurochemical adaptations within this region may be relevant to the behavioral consequences of prolonged β-adrenergic receptor blockade.

### 3.2. Hypothalamic Neurochemical Changes Associated with Propranolol Treatment

The observed changes in neurotransmitter levels may suggest increased neurotransmitter availability and/or synthesis rather than enhanced neurotransmitter turnover or degradation. However, as tissue neurotransmitter concentrations alone do not allow direct assessment of synthesis, release, reuptake, or degradation, these findings should be interpreted cautiously and do not provide direct evidence of increased neurotransmitter production. The most pronounced monoaminergic alteration involved the noradrenergic system. Propranolol increased hypothalamic noradrenaline (NA) concentrations at 1 and 2.5 mg/kg/day, whereas the MHPG/NA ratio was significantly reduced at all doses examined. MHPG is a major metabolite of noradrenaline, and the MHPG/NA ratio can be used as an indirect index of noradrenergic turnover. The reduction in this ratio therefore suggests altered noradrenergic metabolism following prolonged propranolol exposure.

The simultaneous increase in tissue NA and decrease in the MHPG/NA ratio is particularly noteworthy. This pattern does not indicate a simple suppression of noradrenergic neurotransmission. Rather, it suggests a shift in the relationship between tissue NA availability and its metabolic conversion. One possible explanation is the development of compensatory adaptations during sustained β-adrenergic receptor blockade. Prolonged interference with β-adrenergic signaling may modify regulatory mechanisms controlling catecholamine availability and metabolism, resulting in increased tissue NA despite reduced turnover [4,5,33]. Simultaneously, peripheral β-adrenergic blockade may indirectly affect central neurotransmission through afferent signaling, although this pathway was not directly investigated in the present study.

Altered enzymatic metabolism could represent another explanation for this pattern. Reduced conversion of NA to its metabolites could contribute to the simultaneous elevation of NA and reduction in the MHPG/NA ratio. However, enzyme activity was not directly assessed in the present study, and this possibility remains hypothetical. Importantly, the MHPG/NA ratio should be interpreted as an indirect measure of noradrenergic turnover rather than a direct measure of neuronal firing or synaptic neurotransmitter release.

The functional relevance of these findings is supported by the established role of noradrenaline in arousal, vigilance, and stress responsiveness [4,5,22,33]. Excessive noradrenergic activation has been associated with anxiety-related states and maladaptive stress responses [2,4,5,33]. Thus, the reduction in hypothalamic noradrenergic turnover observed in the present study may represent one of the neurochemical correlates accompanying the reduction in anxiety-like behavior.

Long-term propranolol treatment also affected hypothalamic GABA concentrations. The increase was most pronounced at the lower doses, with significant elevations observed at 1 and 2.5 mg/kg/day. The observed increase in hypothalamic GABA levels following chronic propranolol administration is consistent with previous evidence of enhanced GABAergic activity following prolonged propranolol treatment. Previous studies reported increased GABA synthesis and content in the hypothalamus after chronic propranolol administration, suggesting that modulation of hypothalamic GABAergic neurotransmission may contribute to the central effects of propranolol [34,35]. GABA is the principal inhibitory neurotransmitter in the central nervous system and plays an important role in regulating neuronal excitability, emotional behavior, and stress responses [3,36,37,38]. Alterations in GABAergic signaling have been consistently associated with anxiety and increased vulnerability to stress [2,3,36,37,38,39].

The increase in hypothalamic GABA may therefore be relevant to the behavioral phenotype observed in the EPM. Pharmacological enhancement of GABAergic signaling can reduce neuronal excitability and modulate stress-related neural circuits [3,36,37,38,39]. In addition, our previous work demonstrated anxiolytic-like effects following pharmacological enhancement of GABAergic neurotransmission with tiagabine, a selective GAT1 inhibitor [40]. GAT1 is an important transporter responsible for GABA clearance, and its inhibition increases extracellular GABA availability [41,42,43,44]. Although propranolol is not a GAT1 inhibitor, the present findings raise the possibility that prolonged β-adrenergic receptor blockade may indirectly influence GABAergic regulation.

This interpretation should, however, be treated with caution. The present study quantified GABA concentrations in hypothalamic tissue and did not assess extracellular GABA, synaptic release, receptor activity, or downstream signaling. Increased tissue GABA should therefore be interpreted as evidence of altered regional GABA availability rather than definitive evidence of enhanced functional GABAergic transmission. Moreover, GABAergic signaling can exhibit context-dependent effects, and increased tissue GABA does not necessarily predict a uniform increase in inhibitory activity [38,45].

The dopaminergic system showed a different pattern. Neither dopamine (DA) nor its metabolites DOPAC, HVA, and 3-MT were significantly affected by propranolol treatment. Likewise, the DOPAC/DA and HVA/DA ratios remained unchanged.

The dopaminergic system showed a different pattern from the other neurotransmitter systems examined. Neither dopamine (DA) nor its metabolites, DOPAC, HVA, and 3-methoxytyramine (3-MT), were significantly affected by chronic propranolol treatment. Similarly, the DOPAC/DA and HVA/DA ratios remained unchanged, indicating that the treatment did not produce detectable alterations in the hypothalamic dopaminergic parameters assessed. These findings are consistent with those of Laverty and Taylor (1968) [46], who reported no significant changes in brain dopamine, 3-MT, or HVA concentrations following chronic oral propranolol administration in rats. Fuxe et al. (1976) [47] likewise found that propranolol did not alter dopamine concentrations in several forebrain regions, although dopamine turnover increased in certain subcortical limbic regions while remaining unchanged in others. These observations suggest that the effects of propranolol on dopaminergic neurochemistry may depend on the brain region and the specific parameter examined.

More recent evidence also highlights the importance of experimental context when interpreting dopaminergic responses to propranolol. Stocco et al. (2021) [48], using intracerebral propranolol pretreatment to inhibit brain CYP2D in rats exposed to methamphetamine, reported no significant effect on baseline striatal dopamine concentrations, although dopamine release following methamphetamine administration was enhanced. However, this experimental paradigm differs substantially from the chronic propranolol treatment used in the present study and therefore provides complementary rather than directly comparable evidence. Taken together, our findings suggest that chronic propranolol administration did not significantly alter the hypothalamic dopaminergic indices measured in this study. Nevertheless, unchanged tissue concentrations and metabolite-to-DA ratios do not exclude changes in dopamine release, receptor-mediated signaling, or activity within specific neuronal circuits. Thus, the lack of detectable changes in the measured dopaminergic parameters, in contrast to the effects observed in other neurotransmitter systems, further supports the conclusion that the neurochemical effects of chronic propranolol treatment were selective rather than uniform across the systems examined [49].

Serotonergic signaling was also selectively modified. Importantly, this effect was reflected by an increase in 5-HT levels, whereas neither 5-HIAA levels nor the 5-HIAA/5-HT ratio was altered, indicating that the observed modulation of serotonergic signaling was not accompanied by a detectable change in serotonin turnover. Propranolol increased hypothalamic 5-HT concentrations at 1 mg/kg/day, whereas 5-HIAA concentrations and the 5-HIAA/5-HT ratio were not significantly altered. Thus, the increase in tissue serotonin was not accompanied by a detectable change in the turnover index. Serotonin plays an important role in emotional regulation and stress responsiveness [8,50]. Furthermore, serotonergic signaling within hypothalamic circuits can influence neuroendocrine responses to stress, including regulation of the hypothalamic–pituitary–adrenal axis [21,33].

The increase in hypothalamic 5-HT may therefore represent another component of the neurochemical adaptation associated with prolonged propranolol exposure. Interactions between serotonergic and noradrenergic systems are important for the regulation of emotional behavior, and prolonged modulation of β-adrenergic signaling may influence serotonergic function through adaptations within interconnected neural circuits [4,8,27].

Nevertheless, because serotonin turnover was not altered, the present findings support changes in serotonin availability rather than increased serotonergic turnover. Tissue neurotransmitter levels do not directly reflect synaptic release or functional activity, limiting mechanistic interpretation.

### 3.3. Integrated Interpretation of the Neurochemical and Behavioral Findings

An important feature of the present study is that the anxiolytic-like behavioral phenotype was accompanied by changes in several neurotransmitter systems rather than by a single neurochemical alteration.

Simultaneously, tissue neurotransmitter concentrations do not directly reflect their synthesis, release, reuptake, or degradation, and therefore do not allow definitive conclusions about the underlying mechanisms.

Increased GABA concentrations, reduced noradrenergic turnover, and increased hypothalamic 5-HT levels occurred in animals displaying increased open-arm exploration. GABAergic, noradrenergic, and serotonergic systems all contribute to the regulation of stress responsiveness and emotional behavior [4,27,28,51]. Their concurrent modulation may therefore be relevant to the behavioral effects observed following prolonged propranolol treatment.

The relationship between these systems is likely to be complex. Reduced noradrenergic turnover may reflect altered adrenergic regulation of stress-related circuits, whereas increased GABA availability could favor inhibitory control of neuronal activity. At the same time, changes in serotonin availability may influence emotional processing and adaptation to stress [8,33]. Rather than acting through a single neurotransmitter pathway, long-term propranolol exposure may therefore produce a broader adaptive shift in the neurochemical regulation of hypothalamic stress circuits.

The hypothalamus is particularly relevant in this context because it integrates autonomic, endocrine, and behavioral responses to environmental challenges [21,22,27]. Its interaction with distributed stress- and anxiety-related networks is essential for the coordination of behavioral and physiological responses to stress [52,53,54]. Consequently, neurochemical changes detected within the hypothalamus may contribute to altered stress responsiveness. However, the present findings do not establish that these hypothalamic alterations are causally responsible for the observed behavioral phenotype. They should instead be considered candidate neurochemical correlates that provide a basis for further mechanistic investigation.

The distinction between acute and long-term drug exposure is also important. Previous studies of propranolol have reported different effects on central monoaminergic neurotransmission depending on treatment conditions. Differences in dose, treatment duration, and the timing of neurochemical assessment may account for some of these discrepancies. In the present study, relatively low doses were administered over an extended period, potentially allowing adaptive neurochemical processes to develop. Propranolol readily crosses the blood–brain barrier and can influence neural circuits involved in stress adaptation, emotional regulation, and memory processing [9,10]. The neurochemical profile observed here may therefore reflect sustained central β-adrenergic receptor blockade rather than an immediate pharmacological response.

Our previous observations with tiagabine provide additional context for the GABAergic component of the present findings [40]. However, because propranolol is not a GAT1 inhibitor, the increase in hypothalamic GABA cannot be attributed to direct inhibition of GABA reuptake. Further studies will be required to determine whether prolonged β-adrenergic receptor blockade affects GABA synthesis, metabolism, release, transport, or receptor-mediated signaling. Such mechanisms may be particularly relevant given the established involvement of GABAergic circuits in anxiety regulation [40,42,43,44].

### 3.4. Interpretation of the Cardiovascular Effects of Propranolol

The absence of a significant effect of propranolol on blood pressure in the present study warrants consideration. The doses used (1, 2.5 and 5 mg/kg/day) were selected primarily based on previous preclinical studies investigating chronic central and behavioral effects of propranolol, including anxiety-related effects; notably, 2.5 mg/kg/day for four weeks has previously been used to investigate anxiety-like behavior in adult rats [55]. Cardiovascular responses to propranolol at comparable doses are, however, dependent on the experimental model and treatment duration. For example, chronic treatment with 5 mg/kg propranolol reduced heart rate without lowering blood pressure in DOCA/salt-treated rats [56], whereas repeated administration of 5 mg/kg twice daily for 5 weeks reduced both heart rate and systolic blood pressure in normotensive rats [57]. Importantly, [58] demonstrated that chronic propranolol treatment (1 or 5 mg/kg/day for 12 weeks) lowered blood pressure in spontaneously hypertensive rats, whereas this effect was not observed in normotensive rats; heart rate was not consistently affected.

These findings are particularly relevant to the present study, which used normotensive Sprague–Dawley rats and a substantially shorter treatment period of 33 days. Thus, the absence of a significant blood-pressure effect in our animals is consistent with the possibility that a measurable antihypertensive effect of chronic propranolol depends on both the underlying cardiovascular state and longer-term drug exposure. It therefore should not be interpreted as evidence of ineffective drug administration, but rather considered in the context of the dose, duration of treatment and cardiovascular status of the animals.

### 3.5. Strengths, Limitations, and Future Directions

A major strength of the present study is the integration of behavioral and neurochemical approaches to characterize the central effects of long-term propranolol administration. The combination of the EPM and OF allowed anxiety-related behavior to be evaluated while providing an assessment of potential changes in general locomotor activity. The parallel analysis of neurotransmitter concentrations, metabolites, and turnover indices provided a more comprehensive characterization of the neurochemical consequences of prolonged propranolol exposure.

Another strength is the use of a long-term treatment paradigm with relatively low propranolol doses. This approach is relevant because many experimental studies have focused primarily on acute exposure, whereas sustained administration may induce adaptive responses that are not captured following a single drug administration [23,36,43].

Several limitations should nevertheless be acknowledged. First, only male rats were included. Sex-dependent differences in neurotransmitter regulation, stress responsiveness, and anxiety-related behavior are well documented [59,60,61]. Validation of these findings in females will therefore be important.

Second, because propranolol was administered via drinking water to group-housed animals, individual propranolol intake could not be directly measured. Animals were housed in groups of five and shared a treatment bottle; therefore, the estimated dose represents an average cage-level exposure rather than an individually measured dose. Although water consumption was monitored daily and the propranolol concentration was adjusted according to cage-level fluid intake and body weight, individual differences in drinking behavior could not be excluded.

Third, the neurochemical analyses were based on tissue concentrations and metabolite-to-neurotransmitter ratios. These measures provide information about regional neurochemical status but do not directly assess synaptic release, receptor activation, transporter function, or intracellular signaling. In particular, increased tissue GABA cannot be equated with enhanced synaptic GABAergic transmission. Additionally, the analysis was restricted to the hypothalamus. Anxiety-related behavior depends on a distributed neural network involving the amygdala, hippocampus, prefrontal cortex, and brainstem nuclei [22,23,54,62,63,64,65]. Changes in these regions may occur independently of, or in parallel with, the hypothalamic adaptations observed here. Future studies should therefore determine whether long-term propranolol treatment produces region-specific neurochemical changes across the broader neural network involved in stress and anxiety regulation.

Finally, the present experimental design does not permit causal relationships between the observed neurochemical changes and behavioral effects to be established. Pharmacological or molecular manipulation of GABAergic, noradrenergic, and serotonergic signaling will be necessary to determine whether individual pathways are required for the anxiolytic-like effects of long-term propranolol treatment. Assessment of neurotransmitter transporters, receptor expression, and downstream signaling pathways may further clarify the molecular adaptations associated with prolonged β-adrenergic receptor blockade [41,42,43,44]. Additionally, the rat doses cannot be directly extrapolated to therapeutic doses in humans, and the observed behavioral and neurochemical changes do not establish the mechanisms underlying propranolol’s clinical effects in anxiety disorders.

## 4. Materials and Methods

### 4.1. Experimental Animals and Propranolol Treatment

Forty adult male Sprague–Dawley rats (>3 months old) were obtained from a licensed breeder (Mossakowski Medical Research Institute, Warsaw, Poland; registration no. 004). Following arrival, the animals underwent a 7-day acclimatization/quarantine period before the initiation of propranolol treatment. The animals were randomly allocated to four experimental groups (n = 10 per group): a control group (CON) and three propranolol-treated groups receiving target doses of 1 mg/kg b.w./day (PROP 1), 2.5 mg/kg b.w./day (PROP 2.5), or 5 mg/kg b.w./day (PROP 5). The doses of propranolol were selected based on previous preclinical studies investigating the chronic central and behavioral effects of propranolol, including anxiety-related effects [55]. The selected dose range was therefore intended to provide graded chronic propranolol exposure rather than to reproduce an antihypertensive treatment regimen.

Animals were housed in groups of five per cage with (two cages per treatment group) under standard laboratory conditions at a controlled ambient temperature of 20–24 °C and relative humidity of 55 ± 10%, with a 12 h light/12 h dark cycle. Standard laboratory chow and drinking water were available ad libitum throughout the experimental period. Each cage received its own drinking bottle containing the assigned treatment solution. All cages were provided with environmental enrichment, including plastic shelters, cotton rolls, and nesting material.

Propranolol hydrochloride (Merck KGaA, Sigma-Aldrich, Darmstadt, Germany) was administered continuously via drinking water for 33 consecutive days [9]. The drinking solutions were available ad libitum throughout the treatment period. Control animals received tap water under otherwise identical housing and experimental conditions. Water consumption was recorded daily for each cage. Because animals were housed in groups of five, daily fluid intake was evaluated at the cage level rather than for individual animals. Body weight was measured at several predefined time points throughout the study. The average daily propranolol exposure was estimated based on the volume of water consumed by each cage and the mean body weight of the animals housed in that cage. The concentration of propranolol in the drinking water was adjusted when necessary on the basis of these measurements to maintain the intended average daily exposure of 1, 2.5, or 5 mg/kg b.w./day. This procedure allowed the administered propranolol concentration to be adjusted for changes in fluid consumption and body weight during the treatment period. The calculated dose represents an estimated mean cage-level exposure rather than an individually measured dose.

Treatment was initiated 14 days before the start of behavioral testing and continued throughout the behavioral testing protocol. General health status was monitored throughout the experimental period. To minimize stress associated with experimental procedures, the animals underwent a habituation period beginning one week before treatment, including gentle handling and acclimation to the conditions associated with behavioral testing.

### 4.2. Hemodynamic Measurements

Systolic blood pressure (SBP), diastolic blood pressure (DBP), and heart rate (HR) were measured non-invasively using a CODA tail-cuff system (Kent Scientific, Torrington, CT, USA), based on volume pressure recording (VPR) technology. Cardiovascular parameters were assessed at two time points: before initiation of propranolol treatment (baseline) and two days before the beginning of behavioral testing.

Before each measurement session, animals were habituated to the restraint and measurement procedure to minimize stress-related changes in cardiovascular parameters. Measurements were performed under quiet and stable environmental conditions. Each rat was gently placed in a restrainer, and the tail was positioned on the warming platform. An occlusion cuff and a VPR sensor cuff were placed on the tail according to the manufacturer’s recommendations. The tail was warmed to facilitate adequate peripheral blood flow and detection of the pressure signal.

Following stabilization, repeated measurement cycles were performed, and SBP, DBP, and HR were recorded by the CODA system. The device was calibrated according to the manufacturer’s recommendations. The mean values obtained for each animal were used for subsequent statistical analysis.

### 4.3. Behavioral Analysis

Behavioral tests were performed on separate days, with a 24-h interval between consecutive tests. Animal behavior was recorded using a camera mounted above the apparatus and analyzed using EthoVision XT 10 software (Noldus Information Technology, Wageningen, The Netherlands). Behavioral tests were conducted in a dedicated experimental room without natural light, under diffuse and evenly distributed artificial illumination providing sufficient and uniform visibility for video recording and automated behavioral tracking. The lighting conditions were kept consistent throughout the behavioral recordings, with the experimental area arranged to minimize shadows and variations in image quality. Selected behavioral parameters were additionally verified by a trained experimenter.

#### 4.3.1. Open Field Test

The Open Field Test was used to assess spontaneous locomotor activity and exploratory behavior [31,32]. The apparatus consisted of a square arena, with 40 cm-high walls. The central zone in the Open Field Test was defined as the central 50 × 50 cm area of the 100 × 100 cm arena, corresponding to 25% of the total arena surface. Each animal was placed individually in the center of the arena and allowed to explore freely for 5 min.

The following parameters were evaluated: total distance traveled, movement velocity, time spent in movement, time spent in the central zone, frequency of entries into the central zone, climbing.

The maze was thoroughly cleaned with 10% ethanol after each animal to minimize the influence of residual olfactory cues on subsequent trials.

#### 4.3.2. Elevated Plus Maze

Anxiety-like behavior was evaluated using the Elevated Plus Maze (EPM), one of the most widely used behavioral paradigms for assessing anxiety in rodents [29,30,32].

The apparatus consisted of two open arms and two closed arms connected by a central platform. Each arm measured 30 × 5.5 cm. Closed arms were surrounded by 17 cm-high walls. The maze was elevated 63 cm above the floor. Each rat was placed individually on the central platform facing an open arm. The animals were allowed to explore the maze freely for 5 min.

The following variables were recorded: latency to first entry into the open arm, time spent in open arms, time spent in closed arms, number of entries into open arms and total time spent in movement. An arm entry was defined as the placement of all four paws inside an arm of the maze.

Following each trial, the maze was wiped with 10% ethanol to minimize potential olfactory cues between animals.

### 4.4. Tissue Collection

At the end of the experiment, animals were euthanized by decapitation performed by trained personnel. Decapitation without prior anesthesia, as specified in the experimental protocol approved by the Local Ethics Committee for Animal Experiments, was performed to minimize potential confounding effects of anesthetic agents on brain neurochemical measurements, which were among the primary outcomes of the study.

The hypothalamus was rapidly dissected on ice, weighed, and stored at −80 °C until subsequent neurochemical analyses.

### 4.5. Quantification of Monoamines and Their Metabolites

Quantification of monoamines and their metabolites was performed using a previously published and validated chromatographic method [66,67].

Tissue samples were homogenized in 1 mL of ice-cold 0.1 N perchloric acid containing 0.05 mM ascorbic acid. Homogenates were centrifuged at 13,000× *g* for 15 min at 4 °C. The supernatants were filtered through 0.2 μm membrane filters. Monoamines and their metabolites were determined using HPLC with electrochemical detection (HPLC–ED). The HPLC system consisted of an automatic autosampler injector (LaChrom L-7250; Hitachi, Tokyo, Japan, pump (Mini-Star K-500; Knauer, Berlin, Germany), electrochemical detector (L-3500A; Merck-Recipe, Munich, Germany) with a glassy-carbon working electrode. The A 20 μL aliquot of each sample was injected into the chromatographic system. Separation was carried out on a reversed-phase C18 column (250 × 4 mm, 5 μm particle size). The flow rate was set at 0.8 mL/min. The mobile phase consisted of: 32 mM sodium phosphate, 34 mM citric acid, 1 mM octane sulfonic acid, 54 μM EDTA buffer in ultrapure water (18.3 MΩ∙cm) containing 12% methanol. Electrochemical detection was performed at +0.8 V versus an Ag/AgCl reference electrode. The following analytes were quantified: noradrenaline (NA), serotonin (5-HT), dopamine (DA), 3-methoxy-4-hydroxyphenylglycol (MHPG), 5-hydroxyindoleacetic acid (5-HIAA), 3,4-dihydroxyphenylacetic acid (DOPAC), homovanillic acid (HVA), 3-methoxytyramine (3MT). The chromatograms were recorded and integrated by the use of the computerized data acquisition Clarity software (version 8.2; DataApex Ltd., Prague, Czech Republic). The monoamine concentrations in the samples were normalized to tissue wet weight and expressed as pg/mg tissue.

### 4.6. Monoamine Turnover

Monoaminergic activity was estimated using metabolite-to-neurotransmitter ratios. Such indices are commonly used as indirect markers of neurotransmitter metabolism and functional activity [21,22].

Monoamine turnover indices were calculated as metabolite-to-neurotransmitter ratios. Noradrenergic turnover was expressed as the MHPG/NA ratio, dopaminergic turnover as the DOPAC/DA and HVA/DA ratios, and serotonergic turnover as the 5-HIAA/5-HT ratio [22].

### 4.7. Neurochemical Quantification of Amino Acids

The concentrations of selected amino acids in the hypothalamus (gamma-aminobutyric acid (GABA); histidine (HIS); glutamine (GLN); glycine (GLY); aspartic acid (ASP); threonine (THR); arginine (ARG), glutamic acid (GLU), alanine (ALA)) were determined by HPLC with electrochemical detection, using a procedure routinely applied and previously validated in our laboratory [66,67]. The analytical approach was based on previously published protocols for the determination of amino acids in rat brain tissue, with minor modifications adapted to the present study [66].

The chromatography system consisted of an electrochemical detector with a glassy-carbon working electrode, the autosampler (Primade 1210, Hitachi), and the pump (Primade 1110, Hitachi).

Prior to chromatographic analysis, 20 µL of the derivatization reagent was mixed with either amino acid standards or sample supernatants and allowed to react for 15 min at room temperature. The resulting derivatives were separated on a reversed-phase Luna C18(2) column (5 µm, 100 Å, 250 × 4 mm i.d.; Phenomenex, Torrance, CA, USA) at a mobile-phase flow rate of 0.57 mL/min.

Amino acids were quantified using external calibration standards, and analytes were identified according to their retention times and corresponding chromatographic peak characteristics. All amino acid reference standards were obtained from Sigma-Aldrich. Quantitative analysis was performed with Clarity software (version 8.2; DataApex Ltd., Prague, Czech Republic). The concentrations of the analyzed amino acids in brain tissue were normalized to the wet weight of the samples and were expressed as ng/mg of wet tissue. All sample preparation procedures were performed under temperature-controlled conditions to minimize potential degradation of the analytes.

### 4.8. Statistical Analysis

Statistical analyses were performed using TIBCO Statistica version 13.3 (TIBCO Software Inc., Palo Alto, CA, USA). For water consumption and estimated propranolol exposure, the cage was considered the experimental unit because animals within each cage shared the same drinking bottle. Individual animals were considered the experimental unit for body weight, hemodynamic, behavioral, and neurochemical measurements. Data distribution was assessed using the Shapiro–Wilk test, and homogeneity of variance was evaluated using Levene’s test. Hemodynamic parameters were compared between treatment groups using separate one-way analyses of variance (ANOVAs) at each measurement time point. The statistical model used for body weight measurements and the ANOVA models applied to behavioral and neurochemical outcomes are described according to the respective experimental designs. When statistically significant differences were detected, post hoc comparisons were performed using the Newman–Keuls test. Differences were considered statistically significant at *p* < 0.05.

### 4.9. Ethics Statement

All procedures were performed in accordance with Directive 2010/63/EU of the European Parliament and the Polish Act of 15 January 2015 on the protection of animals used for scientific and educational purposes.

The study protocol was approved by the Local Ethics Committee for Animal Experiments in Warsaw, Poland (approval no. WAW2/001/2024, approval date 10 January 2024).

All efforts were made to minimize animal suffering and reduce the number of animals used.

## 5. Conclusions

Long-term propranolol treatment was associated with an anxiolytic-like behavioral phenotype and alterations in hypothalamic neurotransmitter levels. Reduced noradrenergic turnover and increased serotonin and GABA levels may represent neurochemical correlates of these behavioral changes, although their functional significance remains unclear.

These findings suggest that hypothalamic neurotransmitter systems may be involved in the behavioral effects of propranolol, but do not establish the underlying mechanisms. Further studies assessing neurotransmitter release and function, as well as the relevance of these findings to humans, are needed to clarify the mechanisms underlying propranolol-associated behavioral effects.

## Figures and Tables

**Table 1:** Effects of long-term propranolol administration on locomotor activity and exploratory behavior in the Open Field test. Data are presented for control animals (CON) and animals receiving propranolol at doses of 1, 2.5, and 5 mg/kg b.w./day (PROP 1, PROP 2.5, and PROP 5, respectively) (n = 10 per group). Data are presented as mean ± SEM.

Group	Center Zone Entry (Frequency)	Time Spent in the Center Zone (s)	Time Spent Moving (s)	Total Distance Traveled (cm)	Mean Movement Velocity (cm/s)	Climbing (Frequency)
CON	5.5 ± 1.3	46.1 ± 5.9	201.5 ± 13.5	2336.8 ± 213.0	8.0 ± 0.7	14.5 ± 1.8
PROP 1	2.5 ± 0.8	31.7 ± 5.6	192.6 ± 16.1	2056.3 ± 211.9	7.1 ± 0.7	15.7 ± 1.8
PROP 2.5	4.0 ± 1.9	40.1 ± 5.7	194.9 ± 7.4	2289.1 ± 102.8	7.8 ± 0.3	18.0 ± 2.1
PROP 5	2.2 ± 0.5	38.3 ± 8.6	221.7 ± 3.9	2668.6 ± 147.8	8.9 ± 0.5	20.0 ± 2.3

**Table 2:** Effect of long-term propranolol administration on hypothalamic amino acid levels. Values are presented as mean ± SEM (ng/mg tissue, n = 8 per group).

Amino Acid Neurotransmitters	Group	Amino Acid Level (ng/mg Tissue) (Mean ± SEM)
GABA	CON	**844.39 ± 88.52**
	PROP 1	1156.04 ± 53.32 ***
	PROP 2.5	1042.03 ± 16.06 *
	PROP 5	**941.28 ± 27.84** **^▲^**
HIS	CON	24.57 ± 2.19
	PROP 1	29.30 ± 1.90
	PROP 2.5	30.04 ± 2.66
	PROP 5	27.32 ± 2.03
GLN	CON	1377.06 ± 113.70
	PROP 1	1521.96 ± 56.97
	PROP 2.5	1584.56 ± 36.60
	PROP 5	1500.34 ± 48.09
GLY	CON	182.50 ± 16.82
	PROP 1	240.11 ± 7.66
	PROP 2.5	242.24 ± 8.13
	PROP 5	261.35 ± 38.63
ASP	CON	323.91 ± 26.58
	PROP 1	335.77 ± 8.33
	PROP 2.5	373.03 ± 8.08
	PROP 5	348.15 ± 5.13
THR	CON	57.76 ± 6.18
	PROP 1	61.17 ± 4.18
	PROP 2.5	63.96 ± 4.24
	PROP 5	63.15 ± 2.97
ARG	CON	33.08 ± 2.63
	PROP 1	38.03 ± 2.13
	PROP 2.5	35.89 ± 1.18
	PROP 5	36.53 ± 1.81
GLU	CON	1388.258 ± 91.68
	PROP 1	1455.001 ± 28.51
	PROP 2.5	1458.008 ± 25.47
	PROP 5	1528.254 ± 60.62
ALA	CON	57.015 ± 4.54
	PROP 1	61.913 ± 2.42
	PROP 2.5	59.429 ± 2.27
	PROP 5	58.900 ± 1.44

* *p* < 0.05, *** *p* < 0.005 groups vs. the control group (NK); ^▲^ *p* < 0.05 PROP 1 vs. PROP 5 (NK). Bold font indicates significant differences.

**Table 3:** Effect of long-term propranolol administration on hypothalamic monoamine and metabolite levels. Values are presented as mean ± SEM (pg/mg tissue, n = 8 per group).

Monoamine or Metabolite	Group	Monoamine or Metabolite Levels (pg/mg) (Mean ± SEM)
NA	CON	**1112.06 ± 171.01**
	PROP 1	**1685.555 ± 172.98** *
	PROP 2.5	**1616.88 ± 58.49** *
	PROP 5	1340.00 ± 87.64
MHPG	CON	36.39 ± 8.97
	PROP 1	26.63 ± 9.13
	PROP 2.5	19.24 ± 8.52
	PROP 5	12.29 ± 4.95
DA	CON	267.43 ± 59.06
	PROP 1	350.44 ± 39.95
	PROP 2.5	363.33 ± 48.71
	PROP 5	264.37 ± 34.93
DOPAC	CON	46.39 ± 7.59
	PROP 1	71.94 ± 9.73
	PROP 2.5	63.40 ± 17.57
	PROP 5	90.16 ± 16.82
HVA	CON	29.64 ± 8.81
	PROP 1	24.49 ± 4.18
	PROP 2.5	42.49 ± 26.28
	PROP 5	80.30 ± 51.88
3-MT	CON	6.58 ± 1.10
	PROP 1	11.91 ± 2.52
	PROP 2.5	21.54 ± 10.13
	PROP 5	59.128 ± 36.16
5-HT	CON	**598.45 ± 93.92**
	PROP 1	**927.49 ± 112.94** *
	PROP 2.5	679.22 ± 45.15
	PROP 5	726.17 ± 27.22
5-HIAA	CON	446.66 ± 65.39
	PROP 1	554.20 ± 32.76
	PROP 2.5	513.14 ± 30.30
	PROP 5	526.51 ± 29.58

* vs. CON, *p* < 0.05 (NK); Bold font indicates significant differences.

**Table 4:** Monoamine turnover indices (mean ± SEM, n = 8 per group) in the hypothalamus following long-term propranolol administration.

Monoamine Turnover	Group	Monoamine Turnover Indices
DOPAC/DA	CON	0.310 ± 0.10
	PROP 1	0.215 ± 0.03
	PROP 2.5	0.185 ± 0.06
	PROP 5	0.466 ± 0.16
HVA/DA	CON	0.300 ± 0.16
	PROP 1	0.083 ± 0.02
	PROP 2.5	0.139 ± 0.09
	PROP 5	0.385 ± 0.27
5-HIAA/5-HT	CON	0.831 ± 0.10
	PROP 1	0.665 ± 0.08
	PROP 2.5	0.793 ± 0.08
	PROP 5	0.738 ± 0.06
MHPG/NA	CON	**0.044 ± 0.014**
	PROP 1	0.018 ± 0.005 *
	PROP 2.5	0.011 ± 0.006 *
	PROP 5	0.009 ± 0.003 *

* vs. CON. *p* < 0.05 (NK); Bold font indicates significant differences.

## Data Availability

The raw data supporting the conclusions of this article will be made available by the authors upon request.

## Supplementary Materials

The following supporting information can be downloaded at: https://www.mdpi.com/article/10.3390/ijms27219755/s1.
