# Supplementary material for: Long-Term Propranolol Treatment Induces Anxiolytic-like Effects and Alters Hypothalamic Noradrenergic, Serotonergic and GABAergic Neurotransmission
Source: Int J Mol Sci. 2026 Oct 30;27(21):9755. doi: 10.3390/ijms27219755 (PMC13762549; doi:10.3390/ijms27219755)
Supplement: Supplementary file 1 [file ijms-27-09755-s001.zip › ijms-4596362-supplementary.pdf]

	Mean water consumption ml/cage/day							
**Treatment day**	**CON cage 1**	**CON cage 2**	**PROP1 cage 1**	**PROP1 cage 2**	**PROP2.5 cage 1**	**PROP2.5 cage 2**	**PROP5 cage 1**	**PROP5 cage 2**
1	Start of tretmet	Start of tretmet	Start of tretmet	Start of tretmet	Start of tretmet	Start of tretmet	Start of tretmet	Start of tretmet
2	223	188	240	175	234	265	188	268
3	199	215	179	207	247	249	242	172
4	205	186	234	241	183	183	230	170
5	250	194	185	239	191	192	184	210
6	195	200	170	242	213	216	246	231
7	203	197	239	177	176	239	254	176
8	181	235	173	228	184	175	172	174
9	251	251	176	176	228	206	176	211
10	194	234	218	234	220	186	198	238
11	240	219	249	187	181	234	241	250
12	217	174	229	205	214	226	247	177
13	222	214	214	199	229	230	195	207
14	221	223	180	241	224	176	170	253
15	187	212	239	181	232	176	248	183
16	194	204	219	170	235	203	217	177
17	188	185	177	187	252	193	251	234
18	211	223	220	216	237	235	173	207
19	191	222	247	171	174	191	241	205
20	201	178	185	220	188	255	231	219
21	249	242	192	250	196	226	178	188
22	215	182	203	245	221	244	229	177
23	234	235	189	256	206	232	208	207
24	207	174	228	179	171	173	207	255
25	252	207	226	220	221	218	209	252
26	216	249	177	177	187	224	208	232
27	214	228	192	251	254	201	230	193
28	231	222	200	246	180	208	240	185
29	192	196	197	204	235	224	203	214
30	171	190	256	228	256	202	243	235
31	196	183	192	177	210	197	191	239
32	240	252	243	198	196	200	231	191
33	End of treatment	End of treatment	End of treatment	End of treatment	End of treatment	End of treatment	End of treatment	End of treatment

Supplementary Table S1. Daily water consumption in individual experimental cages during the 33-day propranolol treatment.

	CON					PROP1					PROP2.5					PROP5				
	**days 0-6**	**days 7-13**	**days 14-20**	**days 21-27**	**days 28-33**	**days 0-6**	**days 7-13**	**days 14-20**	**days 21-27**	**days 28-33**	**days 0-6**	**days 7-13**	**days 14-20**	**days 21-27**	**days 28-33**	**days 0-6**	**days 7-13**	**days 14-20**	**days 21-27**	**days 28-33**
**mean b.w. (g)**	540	545	553	550	553	527	538	538	537	533	567	584	589	595	585	575	583	591	600	593
**mean water consumption (ml/day per rat)**	41	45	41	44	42	42	41	41	43	43	43	42	42	44	44	43	42	43	42	43
**mean propranolol exposure (mg/kg b.w./day)**	0	0	0	0	0	0.956	0.914	0.914	0.960	0.968	2.275	2.157	2.139	2.218	2.256	4.510	4.322	4.365	4.200	4.350

Supplementary Table S2. Mean body weight, water consumption, and estimated propranolol exposure during the 33-day treatment period.

Body weight was measured at 7-day intervals (days 0, 7, 14, 21, and 28). Mean body weight values assigned to the respective treatment periods were based on these measurements. Water consumption was monitored at the cage level and expressed as mean daily consumption per rat for each treatment period. Estimated daily propranolol exposure (mg/kg b.w./day) was calculated using the nominal drug concentration in drinking water, cage-level water consumption, and the corresponding body weight estimate. Individual water intake and drug exposure were not measured directly.
